# The right temporoparietal junction enables delay of gratification by allowing decision makers to focus on future events

**DOI:** 10.1371/journal.pbio.3000800

**Published:** 2020-08-10

**Authors:** Alexander Soutschek, Marius Moisa, Christian C. Ruff, Philippe N. Tobler

**Affiliations:** 1 Department of Psychology, Ludwig Maximilian University Munich, Munich, Germany; 2 Zurich Center for Neuroeconomics, Department of Economics, University of Zurich, Zurich, Switzerland; 3 Neuroscience Center Zurich, University of Zurich, Swiss Federal Institute of Technology Zurich, Zurich, Switzerland; Oxford University, UNITED KINGDOM

## Abstract

Studies of neural processes underlying delay of gratification usually focus on prefrontal networks related to curbing affective impulses. Here, we provide evidence for an alternative mechanism that facilitates delaying gratification by mental orientation towards the future. Combining continuous theta-burst stimulation (cTBS) with functional neuroimaging, we tested how the right temporoparietal junction (rTPJ) facilitates processing of future events and thereby promotes delay of gratification. Participants performed an intertemporal decision task and a mental time-travel task in the MRI scanner before and after receiving cTBS over the rTPJ or the vertex (control site). rTPJ cTBS led to both stronger temporal discounting for longer delays and reduced processing of future relative to past events in the mental time-travel task. This finding suggests that the rTPJ contributes to the ability to delay gratification by facilitating mental representation of outcomes in the future. On the neural level, rTPJ cTBS led to a reduction in the extent to which connectivity of rTPJ with striatum reflected the value of delayed rewards, indicating a role of rTPJ–striatum connectivity in constructing neural representations of future rewards. Together, our findings provide evidence that the rTPJ is an integral part of a brain network that promotes delay of gratification by facilitating mental orientation to future rewards.

## Abstract 2

Studies of neural processes underlying delay of gratification usually focus on prefrontal networks related to curbing affective impulses. This study reveals that the right temporo-parietal junction improves patience by shifting attention to future outcomes, strengthening the representations of future reward values in the brain.

## Introduction

The ability to delay gratification—sometimes also described as patience or self-control—is thought to be an important predictor for individual life success and health [1,2]. Deficits in this ability are core symptoms of several psychiatric disorders, including addiction and obesity [3,4]. Previous research on the neural basis of delaying gratification mainly focused on how prefrontal networks may reduce affective impulses by modulating value signals in neural reward circuits ([5–7]; but see [8,9]). However, such impulse control is difficult and prone to fail [10], indicating the need for alternative ways of promoting the choice of delayed reward.

We recently provided evidence for a causal link between delay of gratification in intertemporal decisions and neural excitability in the right temporoparietal junction (rTPJ) [11], a brain region that is commonly associated with perspective taking in social cognition [12]. Our finding converged with other demonstrations that rTPJ activity is increased during choice of delayed reward [13,14] and that rTPJ cortical thickness relates statistically to delay of gratification [15]. Thus, the rTPJ appears to be a core node of a network that facilitates delaying gratification by enabling humans to take the perspective of their future selves. In analogy to how the rTPJ may promote prosocial actions by overcoming a focus on the self [16,17], this view suggests that the rTPJ may reduce delay discounting by overcoming a focus on the present. This is consistent with recent theoretical developments aiming to overcome the impulsiveness-oriented standard model by emphasizing the contributions of future orientation and episodic future thinking in delaying gratification [8,9,18–22]. In line with these theoretical assumptions, previous neuroimaging studies linked making patient choices to activation in brain regions involved in prospective future thinking (like anterior cingulate cortex and hippocampus) [23–25]. However, it remains unknown as to how neural mechanisms related to the representation of future events causally affect decision-related computations of subjective reward values assigned to these events.

Here, we reveal the causal neural mechanisms underlying the role of the rTPJ in implementing future-oriented intertemporal choice by testing 3 hypotheses: First, the role of the rTPJ in choosing delayed rewards relates to its more general function for evaluating future events (Hypothesis 1). We derived this prediction from findings that rTPJ activity is enhanced when humans are shifting their mental focus to different time points in the future [26,27]. Second, if the rTPJ strengthens the focus on future outcomes, we expect it to communicate with brain regions encoding the value of delayed rewards (hypothesis 2). In other words, we hypothesize that the rTPJ increases delay of gratification by modulating neural value signals that encode temporally delayed rewards in regions such as the striatum or ventromedial prefrontal cortex (VMPFC) [14,28–30]. Finally, we also consider the more canonical prediction that the rTPJ may facilitate choice of delayed rewards by interacting with regions related to impulse control during intertemporal choice, such as dorsolateral prefrontal cortex (DLPFC) [6,31]. Finding little or no evidence for an interaction between rTPJ and DLPFC would support the notion that the rTPJ might contribute to delaying gratification by a different mechanism than that instantiated by the DLPFC (hypothesis 3). To test these hypotheses, we combined continuous theta-burst stimulation (cTBS) with functional magnetic resonance imaging (fMRI). This allowed us to assess how disrupting rTPJ functioning with cTBS causally changes delaying gratification, the ability to shift attention to future outcomes, as well as communication between the rTPJ and value-encoding–related or impulse-control–related brain regions during intertemporal decision making.

## Results

**Fig 1:**
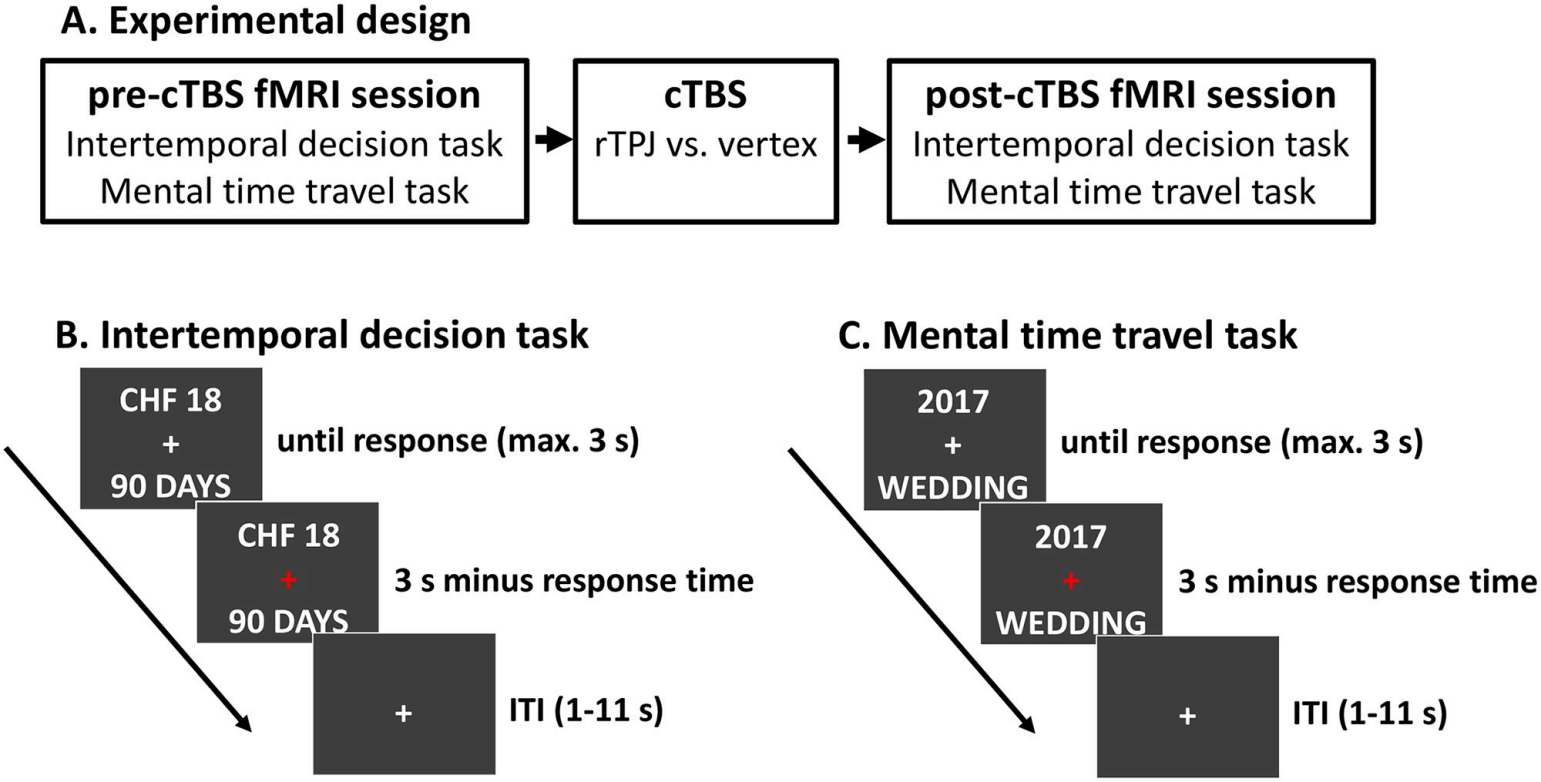
Experimental design and trial structure. (A) Participants performed an intertemporal decision task and a mental time-travel task in a pre-cTBS and a post-cTBS session in the MRI scanner. Prior to starting the post-cTBS session, participants received disruptive cTBS to either the rTPJ or the vertex as control site (between-group design). (B) The intertemporal decision task required choices between an SS option (10 Swiss francs today) and an LL option (10–20 Swiss francs delivered after a delay of 1–180 days). (C) In the mental time-travel task, participants judged whether personal (e.g., “my wedding”) or nonpersonal (e.g., “first man on Mars”) events occurred in the relative past or the relative future as judged either in the present (“2017”) or 8 years in the future (“2025”). cTBS, continuous theta-burst stimulation; rTPJ, right temporoparietal junction.

To test Hypothesis 1 (that rTPJ plays a causal role in choosing delayed rewards by shifting attention of the decision maker to future outcomes), we used cTBS over either the rTPJ or the vertex as control site in a between-group design. Before (pre-cTBS) and after (post-cTBS) receiving cTBS, 60 participants performed an intertemporal decision task as well as a mental time-travel task in the MRI scanner (Fig 1A). In the intertemporal decision task, participants made choices between a smaller-sooner (SS) reward, which was fixed to 10 Swiss francs today, and a larger-later (LL) reward, which ranged from 10 to 20 Swiss francs and was delivered after a delay of 1 to 180 days (Fig 1B). The mental time-travel task required participants to decide whether visually presented personal or nonpersonal events occurred in the relative future or relative past. In addition, participants made these judgements from the point of view of either their current selves or their future selves in 8 years (Fig 1C). For example, the event “submission of Master’s thesis” was in the relative future for the current self of most participants (given that we drew our sample from an undergraduate student population). By contrast, for their future selves in 8 years, this event was likely to be in the past. If the rTPJ promotes delay of gratification by shifting the focus of the decision maker toward future outcomes (hypothesis 1), we expected rTPJ disruption by cTBS to impair both delaying gratification in intertemporal choice and judging events in the future.

### Replication of rTPJ cTBS effects on intertemporal choice

Before testing Hypothesis 1, we ascertained that the new data indeed replicate our previous findings [11] that rTPJ cTBS increases delay discounting. We characterized the specific shape of reward discounting across the associated temporal delay by fitting discount functions to the choices of each participant. The generalized hyperbolic discount model (mean leave-one-out cross-validation information criterion [LOOIC] = 75.6) explained the data better than the one-parameter hyperbolic (mean LOOIC = 88.7) or the quasi-hyperbolic (“beta-delta”) model (mean LOOIC = 85.9). In the winning generalized hyperbolic discount model, the steepness of the discount function is determined by 2 parameters, a discount factor k that is more sensitive to shorter delays, and a scaling parameter s that has been related to subjective time perception [32] and determines the strength of discounting across time, particularly at longer delays [33].

**Fig 2:**
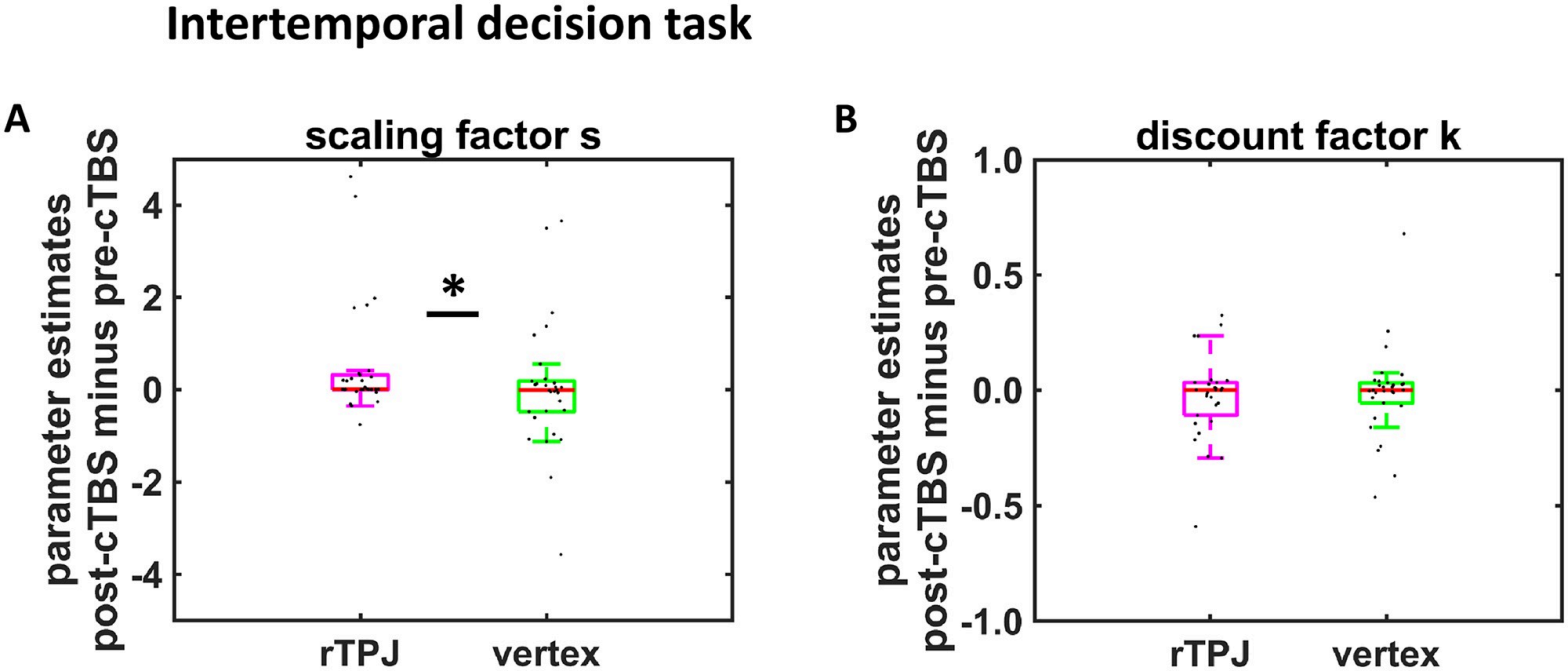
Effects of rTPJ cTBS on intertemporal choice. (A, B) Impact of cTBS (rTPJ versus vertex) on post-cTBS minus pre-cTBS differences in parameter estimates for (A) scaling factor s and (B) discount factor k in generalized hyperbolic discounting. Boxes indicate median (in red) and interquartile range. Black dots show individual parameter estimates. Asterisk indicates significant effect (*p* < 0.05, one-tailed, nonparametric Mann-Whitney U tests. Thus, the significant difference between stimulation groups in (A) is not due to outliers). The underlying data for this Figure can be found in S1 Data. cTBS, continuous theta-burst stimulation; rTPJ, right temporoparietal junction.

Using nonparametric Mann-Whitney U-tests (to account for the skewed distributions of parameters and to remove the impact of outliers), we observed no differential effects of rTPJ versus vertex cTBS effects on post-cTBS minus pre-cTBS changes in k (*Z* = 0.67, *p* = 0.33, one-tailed, effect size r = 0.06). However, rTPJ cTBS increased the time-perception–related scaling parameter s in the post-cTBS relative to the pre-cTBS session more than vertex cTBS did (*Z* = 1.80, *p* = 0.04, one-tailed, r = 0.23) (Fig 2). The cTBS effect on s was robust to correcting for individual baseline differences by dividing post-cTBS minus pre-cTBS difference scores by pre-cTBS baseline values (*Z* = 1.80, *p* = 0.04, one-tailed, r = 0.23). This replicates our previous findings in which cTBS affected mainly parameters sensitive to longer delays. Moreover, as the parameters k and s are potentially related, we assessed whether cTBS lowered also the area under the curve (AUC) of the generalized hyperbolic discount function (which integrates the influences of k and s on behavior). Post-cTBS minus pre-cTBS changes in AUC were significantly more negative after rTPJ cTBS compared with vertex cTBS (*Z* = 1.97, *p* = 0.02, one-tailed, r = 0.25). As smaller AUC indicates more impulsive choice behavior, this result supports the assumption that rTPJ disruption reduces patience. On the worse-fitting one-parameter hyperbolic and quasi-hyperbolic models, there were no significant cTBS effects on any parameter (all *Z* < 1.15, all *p* > 0.12, one-tailed, all r < 0.15). Also, a re-analysis of our old data set (using the same estimation approach as for the current data set) provided converging evidence that rTPJ cTBS increases the time-perception–related scaling parameter in generalized hyperbolic discounting (SI Text). Taken together, the current study is thus the third experiment providing evidence that disrupting rTPJ functioning increases delay discounting for longer delays [11].

It is worth noting that cTBS did not induce higher decision noise: For none of the previously described models did we observe significant cTBS effects on post-cTBS minus pre-cTBS differences in inverse temperature parameters (all *Z* < 1.30, all *p* > 0.19, all r < 0.17). There was also no evidence that participants experienced TPJ cTBS as more aversive than vertex cTBS (*t*(58) < 1, *p* > 0.88). Thus, unspecific noise or mood effects cannot explain our finding that the TPJ plays a causal role in promoting delay of gratification.

### rTPJ cTBS impairs processing of future versus past events

We started our test of Hypothesis 1 by assessing cTBS effects on the mental time-travel task. This task differentiates between events in the relative past versus relative future, between the perspective from which events are evaluated (i.e., from one’s current or future perspective), as well as between personal and nonpersonal types of events. Based on existent imaging data [27], we assessed whether the rTPJ is causally involved in making such time-related judgements.

**Fig 3:**
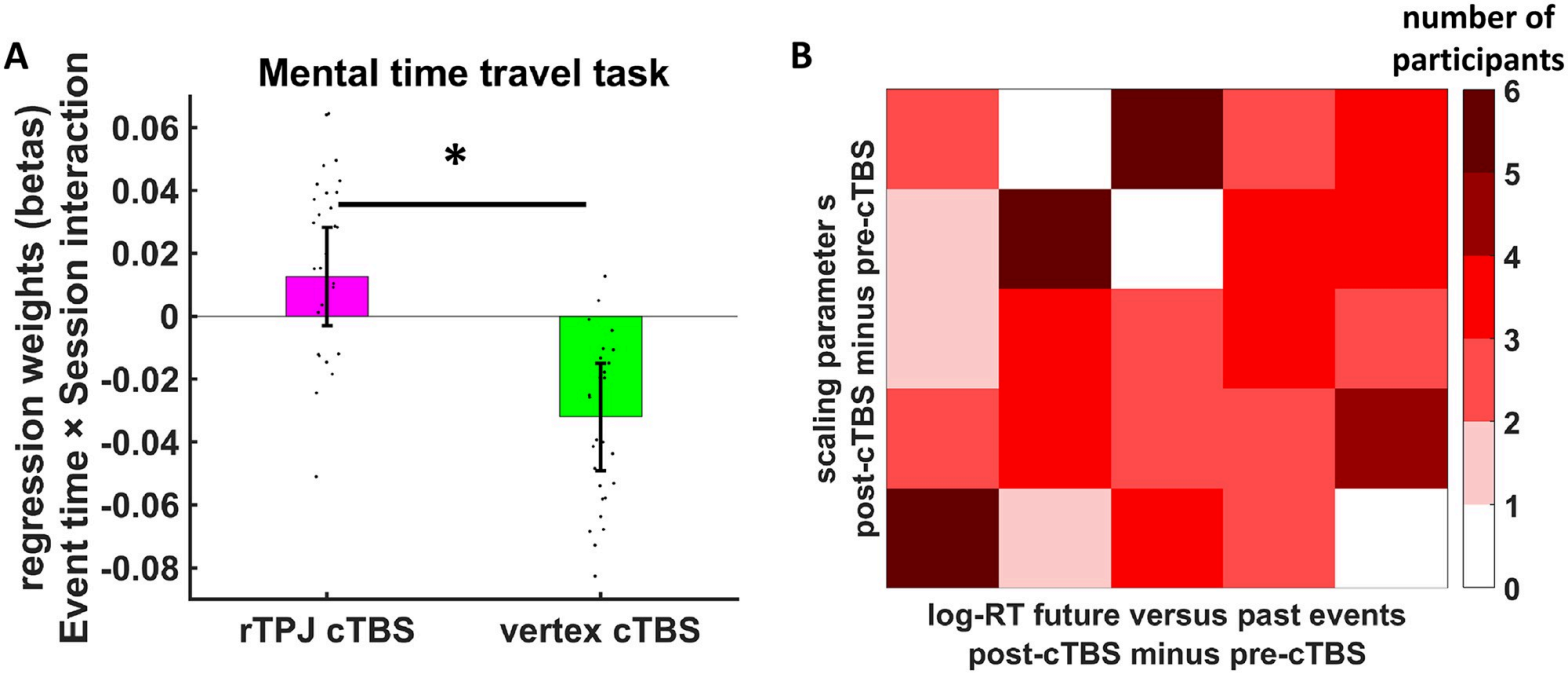
Effects of rTPJ cTBS on mental time traveling. (A) rTPJ, relative to vertex, cTBS, increased the post-cTBS minus pre-cTBS difference in RT (log-RT) for judging future relative to past events. Error bars indicate standard error of the mean. Black dots show individual parameter estimates (based on random effects estimates). Asterisks indicate significant effects (*p* < 0.05). (B) Heatmap illustrating the correlation between cTBS-induced post-cTBS minus pre-cTBS changes in the mental time-travel task (binned log-RT difference between events in the relative future and relative past) and the intertemporal choice task (binned scaling parameter s from generalized hyperbolic discounting). Darker colors indicate a higher number of participants in a given cell. Note that the darker colors locate primarily around the diagonal, illustrating the correlation between the cTBS effects in the two tasks. The underlying data for this Figure can be found in S1 Data. CHF, Swiss francs; cTBS, continuous theta-burst stimulation; fMRI, functional magnetic resonance imaging; RT, response time; rTPJ, right temporoparietal junction.

To test this hypothesis, we analyzed log-transformed response times (RTs) in the mental time-travel task with a mixed generalized linear model (MGLM) that regressed RTs on predictors for cTBS (rTPJ versus vertex), session (post-cTBS versus pre-cTBS), run (first versus second run within a session), perspective (now versus future), event type (personal versus nonpersonal), event time (relative past versus relative future), and all interactions between these factors. Our analysis revealed significant main effects of perspective, event type, and event time (all beta > 0.033, *t* > 2.70, *p* < 0.007), suggesting that participants were slower (1) when judging events from a future relative to the current perspective, (2) for nonpersonal relative to personal events, and (3) for events in the relative future compared to the relative past. In the vertex group, this RT slowing for relative future compared with relative past events was reduced in the post-cTBS relative to the pre-cTBS session, presumably due to practice effects, session × event time interaction (beta = −0.032, *t*(3746) = 1.98, *p* = 0.048). Importantly, the significant cTBS × session × event time interaction (beta = 0.045, *t*(3705) = 1.96, *p* = 0.05) showed that rTPJ versus vertex cTBS differentially changed the ability to judge future—relative to past—events in the post-cTBS relative to the pre-cTBS session: compared with vertex cTBS, rTPJ cTBS slowed responses to events in the relative future compared to the relative past (Fig 3A; we note, though, that a further MGLM selectively for the rTPJ group showed no significant session × event time interaction (beta = 0.013, *t*(2021) = 0.81, *p* = 0.42). Moreover, the impact of cTBS on future event processing tended to be more pronounced for judgements that participants made from their current than their future perspective, cTBS × session × event time × perspective interaction (beta = −0.059, *t*(3949) = 1.85, *p* = 0.06), as well as in the first relative to the second run of a session, cTBS × session × event time × run interaction (beta = −0.081, *t*(6198) = 2.54, *p* = 0.01). The latter finding might be explained by the waning nature of stimulation effects. Taken together, our results show that cTBS over rTPJ—relative to vertex—both increases delay discounting at longer delays in the intertemporal decision task and impairs the ability to process future events in the mental time-travel task.

The common effect of cTBS on both tasks already suggests that delay discounting and making temporal judgements are implemented by a common neural network. To test this more directly, we investigated the relation between the stimulation effects on delay discounting and mental time-travel tasks. We found that post-cTBS minus pre-cTBS changes in the ability to judge relative future compared with relative past events were significantly correlated with post-cTBS minus pre-cTBS changes in the scaling parameter s from the best-fitting generalized hyperbolic discount model, Spearman’s rho = 0.17, *p* = 0.03, one-tailed (Fig 3B). That is, the more cTBS made participants slower in judging relative future versus relative past events, the more it also made them impulsive for long delays in the delay-discounting task. This relationship between cTBS effects on delay discounting and temporal judgements provides further support that the rTPJ is a common neural substrate for processing future events and the discounting of delayed rewards in intertemporal choice.

### rTPJ–striatum interactions modulate neural value signals during intertemporal choice

**Fig 4:**
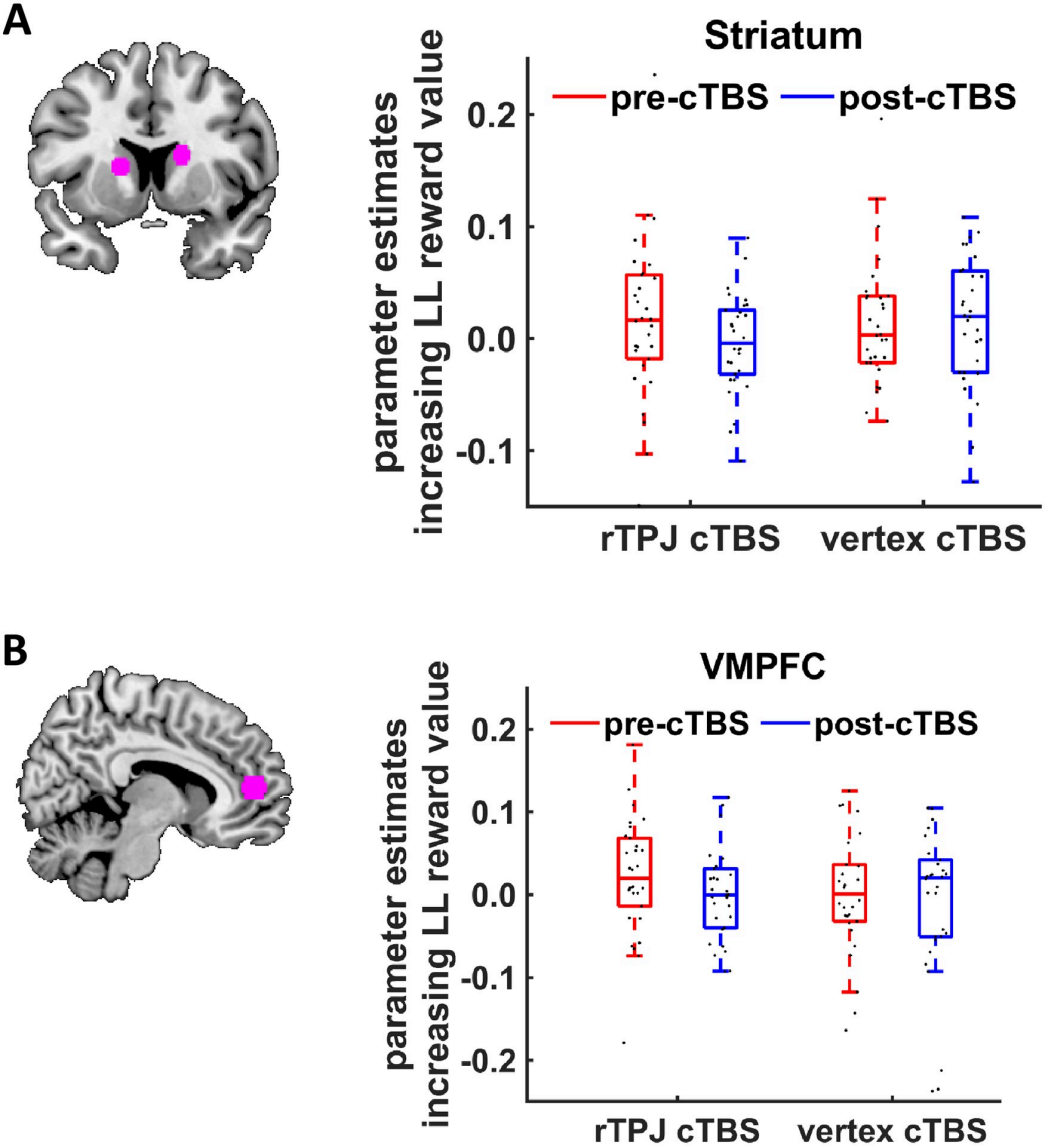
Neural encoding of delayed (future) reward value in the intertemporal decision task. Illustration of ROIs for (A) striatum and (B) VMPFC based on [31]. We observed no cTBS effects on value-related striatum or VMPFC activation. Boxes indicate median (in red) and interquartile range. Black dots show individual parameter estimates. The underlying data for this Figure can be found in S1 Data. cTBS, continuous theta-burst stimulation; LL, larger-later; ROI, region of interest; VMPFC, ventromedial prefrontal cortex.

The behavioral results suggest a crucial role of the rTPJ for delaying gratification in intertemporal choice by strengthening the mental focus on self-related future outcomes. Hypothesis 2 posits that the rTPJ might thus communicate with regions implementing neural value signals during intertemporal choice. To test this prediction, we first computed a generalized linear model (GLM) that examined neural representations of the subjective value of delayed rewards, as given by the individual discount functions based on generalized hyperbolic discounting as the best-fitting model showing significant cTBS effects. Because previous studies found discounted value of LL rewards to be represented in striatum and VMPFC [14,30,31,34], we tested whether activation in these areas correlated with the subjective value of delayed rewards. For this purpose, we used signals extracted from regions of interest (ROIs) for striatum and VMPFC pre-defined based on a recent meta-analysis of brain activity during delay-discounting tasks [31] (Fig 4). As a robustness check, we also employed alternative ROIs based on a meta-analysis on general value coding [28] and a canonical study on the neural correlates of temporally discounted reward value [14]. We interpret cTBS effects only if they are statistically significant for all ROI definitions. Using the striatum ROI from the delay-discounting meta-analysis, we found the expected positive correlation between subjective LL reward value and activation in the striatum (*Z* = 2.06, *p* = 0.04, r = 0.27) (Fig 4A), replicating previous findings [14,30]. However, there were no significant cTBS effects on post-cTBS minus the pre-cTBS changes in value-related activation in the striatum for any of the 3 employed ROIs (see earlier) (all *Z* < 1.51, all *p* > 0.26, Bonferroni-corrected, all r < 0.20). VMPFC activation showed no correlation with subjective value when using the ROI from the delay-discounting meta-analysis (*Z* = 1.41, *p* = 0.16, r = 0.18) (Fig 4B), and there was no evidence that rTPJ (relative to vertex) cTBS significantly changed post-cTBS minus pre-cTBS changes in value-related signals in the VMPFC ROI (*Z* = 0.95, *p* = 0.68, Bonferroni-corrected, r = 0.12) nor for 2 alternative definitions of the VMPFC ROI (both *Z* < 0.92, both *p* > 0.70, Bonferroni-corrected, both r < 0.12).

In addition to the ROI-based approach, we also performed exploratory whole-brain analyses testing for neural correlates of discounted LL reward value in the pre-cTBS session and for cTBS effects on post-cTBS minus pre-cTBS differences. In the pre-cTBS session, discounted LL reward value correlated with activation in the left and right posterior parietal cortex, left middle frontal gyrus, and left fusiform gyrus (whole-brain family-wise error corrected at the cluster level; S1 Table). However, when testing for effects of rTPJ versus vertex cTBS on post-cTBS minus pre-cTBS changes, there were no significant differences between rTPJ and vertex cTBS groups in any brain region, even at lenient exploratory statistical thresholds (no significant clusters at *p* < 0.005, uncorrected, minimum cluster size k ≥ 10). Thus, there was no evidence that rTPJ cTBS changed neural activation related to computations of delayed reward values.

**Fig 5:**
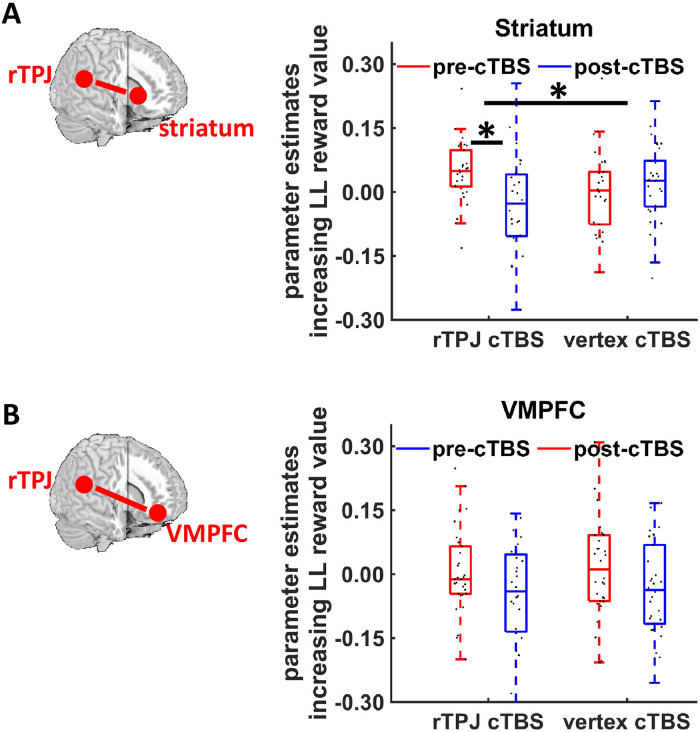
Effects of rTPJ cTBS on connectivity between rTPJ and (A) striatum as well as (B) VMPFC. (A) Disrupting rTPJ with cTBS reduced LL-value–related coupling between rTPJ and striatum. Coupling was significantly reduced in the post-cTBS relative to the pre-cTBS session only following rTPJ, not vertex, cTBS. This finding suggests that the rTPJ modulates neural value signals during intertemporal choice. (B) We observed no significant cTBS effect on VMPFC activation. Black dots show individual parameter estimates. Asterisks indicate significant effects (*p* < 0.05). The underlying data for this Figure can be found in S1 Data. cTBS, continuous theta-burst stimulation; LL, larger-later; rTPJ, right temporoparietal junction; VMPFC, ventromedial prefrontal cortex.

We next investigated the neural mechanism through which the rTPJ-mediated focus on future outcomes may increase the value of these future events. We did so by testing whether rTPJ cTBS affected the communication between rTPJ and areas computing value during intertemporal choice (Hypothesis 2), in general concordance with previous demonstrations of decision-specific changes in functional coupling of rTPJ with value-related areas [17,35–37]. In a psycho-physiological interaction (PPI) analysis, we identified areas showing rTPJ-cTBS–induced changes in LL-value–related functional coupling with the stimulated rTPJ (using a 16-mm-diameter sphere around the rTPJ stimulation site as seed region) (PPI-1). For this analysis, we again extracted parameter estimates from striatum and VMPFC ROIs for the PPI interaction term (rTPJ activation multiplied with subjective LL reward value). In the striatum ROI based on the delay-discounting meta-analysis (Fig 5A), we found that post-cTBS minus pre-cTBS changes in LL-value–related coupling with the rTPJ were differentially affected by rTPJ- versus vertex-cTBS (*Z* = 2.47, *p* = 0.03, Bonferroni-corrected, r = 0.32). This interaction arose from significantly reduced LL-value–dependent connectivity in the post-cTBS relative to the pre-cTBS session in the rTPJ cTBS group (*Z* = 2.60, *p* = 0.009, r = 0.34), whereas no such difference was found for the vertex cTBS group (*Z* < 1, *p* = 0.56, r = 0.08). We note that the cTBS effects on post-cTBS minus pre-cTBS changes in TPJ–striatum connectivity were also present in more ventral striatum ROIs coding specifically the value of delayed rewards (*Z* = 2.29, *p* = 0.04, Bonferroni-corrected, r = 0.30), and showed a trend-level effect for the general value-coding striatum ROI [14,28] (*Z* = 2.12, *p* = 0.07, Bonferroni-corrected, r = 0.27). Thus, rTPJ-cTBS disrupted value-related connectivity between rTPJ and striatum during intertemporal decisions.

A different picture emerged for connectivity between rTPJ and VMPFC: we found no cTBS effects on post-cTBS minus pre-cTBS changes in the VMPFC ROI based on the delay-discounting meta-analysis (*Z* = 0.16, *p* = 1, Bonferroni-corrected, r = 0.02) nor for 2 alternative definitions of the VMPFC ROI (both *Z* < 1.04, both *p* > 0.60, Bonferroni-corrected, both r < 0.14) (Fig 5B). Additional exploratory whole-brain analyses provided no evidence that other brain regions may have shown enhanced functional connectivity with rTPJ in the pre-cTBS session (thresholded at *p* < 0.001, minimum cluster size k ≥ 10, uncorrected), or that post-cTBS minus pre-cTBS changes in rTPJ connectivity significantly differed between rTPJ and vertex cTBS groups (S2 Table). Taken together, our results support Hypothesis 2 in that the rTPJ promotes delay of gratification by communicating with brain regions that encode the subjective value of future rewards during intertemporal choice.

### rTPJ cTBS reduces value-related DLPFC activation

**Fig 6:**
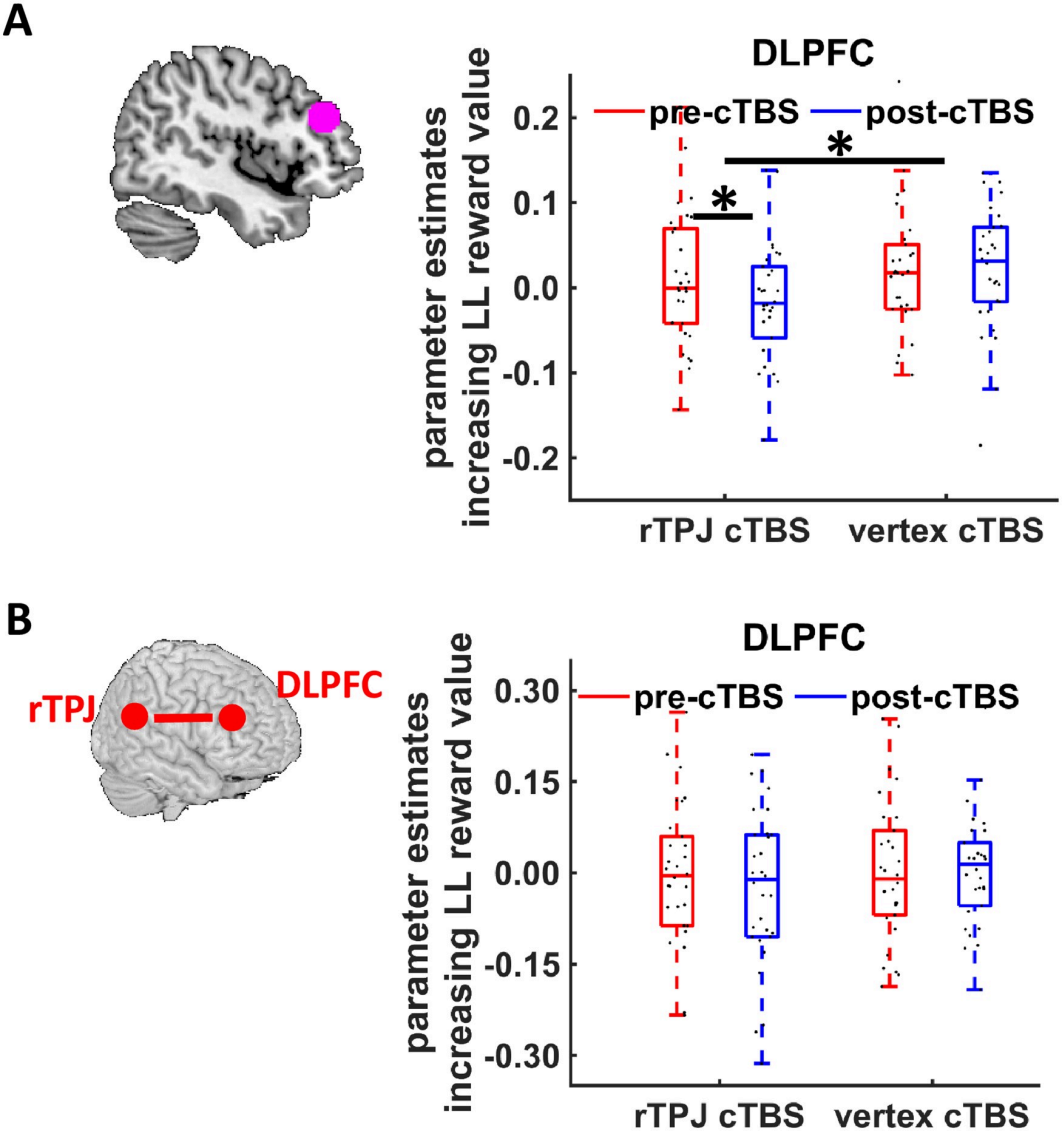
Effects of rTPJ cTBS on (A) value-related DLPFC activation and (B) connectivity between rTPJ and DLPFC. (A) Neural encoding of delayed (future) reward value in the DLPFC. Parameter estimates for value-related DLPFC activation were significantly reduced following rTPJ, not vertex, cTBS. (B) There was no evidence for cTBS effects on functional connectivity between rTPJ and DLPFC activation. Boxes indicate median (in red) and interquartile range. Black dots show individual parameter estimates. Asterisks indicate significant effects (*p* < 0.05). The underlying data for this Figure can be found in S1 Data. cTBS, continuous theta-burst stimulation; DLPFC, dorsolateral prefrontal cortex; LL, larger-later; rTPJ, right temporoparietal junction.

Next, we tested whether the rTPJ promotes delay of gratification independently of the prefrontal control network (Hypothesis 3). GLM-1 revealed that subjective discounted reward value correlated with enhanced DLPFC activation in the pre-cTBS session (S1 Table), replicating previous findings [14]. Next, we assessed cTBS effects on value-related DLPFC activation by extracting parameter estimates of GLM-1 from a pre-defined region in DLPFC, based on the same meta-analysis of temporal discounting [31] used for definition of all ROIs. We observed a significant cTBS effect on post-cTBS minus pre-cTBS changes in value-related DLPFC activation (*Z* = 2.20, *p* = 0.03, r = 0.28) (Fig 6A). DLPFC activation was reduced in the post-cTBS relative to the pre-cTBS session after rTPJ cTBS (*Z* = 1.94, *p* = 0.05, r = 0.35), in contrast to after vertex cTBS (*Z* = 1.10, *p* = 0.20, r = 0.20). Similar results emerged in analyses using ROIs based on other studies relating DLPFC to impulse control in intertemporal choice [6,38] (all *Z* > 1.99, all *p* < 0.05, all r > 0.25). These findings suggest that the rTPJ implements delay of gratification in interaction with the prefrontal control system, contrary to Hypothesis 3.

Finally, we tested for potential coupling of rTPJ with DLPFC by extracting parameter estimates for the DLPFC ROI from the pre-cTBS and post-cTBS sessions of the PPI analysis reported above (PPI-1). The two cTBS groups did not differ with regard to the changes in rTPJ-DLPFC coupling between pre-cTBS and post-cTBS session for all DLPFC ROIs (all *Z* < 0.70, all *p* > 0.48, all r < 0.07) (Fig 6B). Thus, our connectivity analyses provided little evidence that rTPJ cTBS changed rTPJ-DLPFC connectivity.

## Discussion

Forgoing short-term gratification for the sake of long-term rewards is crucial for goal-directed behavior, a process that is commonly thought to be implemented by a prefrontal control network [6]. Here, we provide functional and neural evidence for a causal role of the rTPJ and its interactions with value-coding areas in delaying gratification. Our results suggest a neural link between delaying gratification and shifting the mental focus of decision makers to the future: disrupting rTPJ functioning impaired both the ability to evaluate future events in the mental time-travel task and the capacity to delay gratification (though only in the best-fitting generalized hyperbolic discount model in which cTBS affected the scaling parameter related to time perception [32]). This suggests that the rTPJ might implement delay of gratification in intertemporal choice by enabling decision makers to focus on future events. Our findings thus reveal the causal brain networks involved in computing the subjective value of future rewards, providing neural evidence for theoretical accounts incorporating the role of future perception in intertemporal choices [8,9,18–22]. Previous imaging studies provided correlative evidence for a role of anterior cingulate cortex and its interactions with hippocampus and amygdala for future orientation in delay of gratification [23–25]. However, the link between these activations and the computation of subjective reward values remained unclear. The current results indicate that the rTPJ may play a crucial role in this respect, by causally influencing the computation of future reward values in the striatum to promote future-oriented behavior.

Our findings expand the role of the rTPJ in perspective taking, which previously has been mainly studied in social situations [12,16,17]. By contrast, we show that the rTPJ also causally contributes to processing future events. In analogy to encoding the mental states of others in social interactions, the rTPJ appears to be a key part of a network enabling humans to encode future goals and desires, an essential precondition for future-oriented behavior [26,27]. Moreover, our results are at variance with imaging findings reporting no significant differences in rTPJ activation between processing of relative future and relative past events [26,27]. Thus, the mental time-travel task data provide first causal evidence that shifting mental focus to the future might in fact rely more strongly on rTPJ activation than shifting it to the past.

The idea that the rTPJ strengthens the focus on future outcomes in temporal discounting implies that the rTPJ should communicate with brain regions encoding the value of these future outcomes. Crucially, combining cTBS with functional imaging allowed us to test this prediction by identifying the neural network mechanisms by which the rTPJ orchestrates future orientation during intertemporal choice. The observed rTPJ cTBS-induced reduction of functional connectivity with the striatum, and of willingness to wait in the delay-discounting task, provide evidence for a causal role of rTPJ–striatum connectivity in delaying gratification by enhancing the focus on future outcomes. The striatum is part of the reward processing network [28,29] and thought to encode the subjective value of delayed rewards [14,30,38,39]. Our findings therefore suggest that neural representations of future reward values are strengthened by means of connectivity between rTPJ and value-coding brain regions, mediated by anatomical connections between rTPJ and the putamen [40].

It is worth noting that similar connectivity between rTPJ and neural reward regions operates in the domain of social decision making, where the rTPJ increases neural signals associated with decision values in strategic interactions [36,37] as well as with the value of costly giving [17,35]. In situations in which an individual has to decide whether to share money with others, the neural reward system may represent the value of sharing [41], while the rTPJ may allow taking the perspective of others [12]. Increased connectivity between the rTPJ and the neural reward system during costly giving could indicate that taking the perspective of others strengthens the value of sharing [17]. The rTPJ may thus perform related computations in intertemporal and interpersonal decision making that serve to determine and enhance the subjective value of both temporally and socially more distant reward options.

While our data indicate that the rTPJ implements future-oriented behavior by communicating with striatal subregions involved in value computation, the present findings also suggest that disrupting rTPJ functioning reduces value-related brain activation in the DLPFC. In intertemporal choice, DLPFC is thought to implement self-control by enhancing the value of LL relative to SS rewards in the neural value system [7,38]. Increased DLPFC activity may facilitate choice of delayed rewards by encoding the goal of maximizing the long-term payoff in working memory [31,42]. It is tempting to speculate that a stronger focus on future goals and outcomes, as implemented by rTPJ, may thus increase the strength of the representation of long-term goals in DLPFC. Interestingly, we observed no stimulation effect on rTPJ–DLPFC connectivity (contrary to value-related DLPFC activation itself), and in fact (to the best of our knowledge) there is no evidence for direct fiber connections between rTPJ and DLPFC [40]. rTPJ disruption might therefore have affected value-related DLPFC activation indirectly via other regions involved in value processing that receive input from rTPJ, like striatum, ventrolateral PFC, or insula [40]. We also note that we restricted the ROI analysis to striatum, VMPFC, and DLPFC given our a priori hypotheses for these regions, but this leaves open the possibility that other brain regions may show cTBS effects that are not detected in our whole-brain analysis but may still be significant in other hypothesis-guided ROI analyses. We therefore refrain from concluding that the tested regions are the only ones that are causally affected by rTPJ cTBS in intertemporal choice.

The presently characterized neural mechanism implementing future orientation may also play a role in the symptoms of psychiatric disorders with self-control deficits. Patients with addiction, for example, show steeper temporal discounting of delayed rewards than healthy controls, which is commonly attributed to impulse control deficits [4]. However, the evidence for a crucial role of future orientation for delaying gratification suggests that the impulsivity deficits in these disorders might have more than one cause. For example, smokers are temporally more present-oriented than nonsmokers [43], consistent with the view that reduced future orientation might contribute to impulsivity in addiction as well. While previous neural interventions in addiction mainly focused on the fronto-striatal impulse control network [44], the rTPJ might thus provide a promising alternative target for neuromodulatory treatments of impulsivity in addiction.

To conclude, our findings open a novel approach for increasing delay of gratification via rTPJ-mediated processing of personal future decision outcomes. On the neural level, this enhanced focus on future outcomes is reflected by the rTPJ communicating with neural reward signals that encode the value of future rewards.

## Materials and methods

### Ethics statement

The study protocol was approved by the Research Ethics Committee of the canton of Zurich (protocol number: 2010–0326) and conducted according to the principles expressed in the Declaration of Helsinki. All volunteers gave written informed consent prior to their participation.

### Participants

Sixty volunteers (37 female, M_age_ = 23.4, SD_age_ = 2.4), recruited through the local participant pool, were randomly assigned to one of the 2 stimulation groups (rTPJ or vertex). To replicate our previous effect of rTPJ cTBS on delay discounting [11], we performed a power calculation that suggested that a minimum of 28 participants per stimulation group was needed in order to find a significant effect (alpha = 5%, one-tailed) with a power of 80%, assuming the effect size observed in our previous study. Participants received 110 Swiss francs for their participation and a monetary bonus that depended on their choices (see below).

### Task design

Participants performed 2 tasks in the MRI scanner: an intertemporal decision task and a mental time-travel task. In the intertemporal decision task, participants chose between an SS reward that was fixed to 10 Swiss francs today and an LL reward that ranged from 10 to 20 Swiss francs and that was delivered after a delay of 1 to 180 days. We fully crossed 9 reward levels (10, 11, 12, 13, 14, 15, 16, 18, and 20 Swiss francs) with 9 different delays (1, 5, 10, 20, 40, 60, 90, 120, and 180 days), resulting in a total of 81 reward–delay combination trials (which were presented in random order and repeated twice per session). As the amount for the SS reward option was fixed, only the amount and delay for the LL reward were presented on the screen above and below a central fixation cross, respectively. The offer was presented for a period of 3 seconds during which participants had to indicate their choice by pressing the left or right response key of a scanner-compatible button box (with the assignment of keys to SS and LL reward choices counterbalanced across participants). Once a response was made, the central fixation cross turned red for the remaining time of the 3-second response interval. The next trial started after a variable inter-trial interval (ITI). The minimum ITI was 1 second and was jittered with a Poisson distribution (mean = 2 seconds, maximum = 11 seconds).

In the mental time-travel task, participants judged whether personal (e.g., “30th birthday”) or nonpersonal (e.g., “first man on Mars”) events were either in the relative past or in the relative future. Participants had to make these judgements from the point of view of either the current year (indicated by the cue “2017,” as data were collected in the year 2017) or 8 years in the future (indicated by the cue “2025”). In each session, 9 events were presented per cell of the design (personal/nonpersonal × future/past × current/future perspective) in random order, resulting in a total of 72 events. One-half of participants pressed the left key for events in the relative future and the right key for events in the relative past, and the other half of participants used the inverse key-to-response assignment. As for the intertemporal decision task, participants had to provide a response during the stimulus presentation period of 3 seconds. After each response, the central fixation cross turned red and stayed on the screen together with the perspective cue and event for the remaining length of the decision period, followed by a variable ITI of 1–11 seconds (again sampled from a Poisson distribution with mean = 2 seconds).

### Procedure

Participants performed the experimental tasks in a pre-cTBS and a post-cTBS session, with each session comprising 2 runs. One run lasted 12 minutes and included 3 mini-blocks (each containing 27 trials) of the intertemporal decision task and 3 mini-blocks (each containing 12 trials) of the mental time-travel task in randomized order. Within each mini-block, trials were presented in randomized order. Thus, a total of 81 trials of the intertemporal decision task and 36 trials of the mental time-travel task were presented per run.

At the end of the experiment, a single trial of the intertemporal decision task was randomly chosen and implemented in addition to the basic payment of 110 Swiss francs. If participants had chosen the SS option in the selected trial, they obtained the corresponding amount immediately. If participants had chosen the LL option, they received the specific amount after the corresponding temporal delay via mail.

### cTBS

Participants were stimulated either over the rTPJ (30 participants) or over the vertex (30 participants) with a standard cTBS protocol [45]. Participants received cTBS with an MR-compatible coil (Magventure MRi-B91) on the bed of the MR scanner over the coordinates identified with Brainsight (see below). The stimulation site and coil orientation (see below) were marked on a latex cap fixed on the head. For cTBS, bursts of 3 stimuli at 50 Hz were repeated with a frequency of 5 Hz for 40 seconds, resulting in a total of 600 pulses. Stimulation intensity was set to 80% of the active motor threshold. Motor threshold corresponded to the lowest TMS pulse intensity required to elicit a motor-evoked potential larger than 200 μV from the contralateral first *dorsal interosseous* muscle on more than 5 out of 10 trials while the participant maintained a contraction of about 20% maximum force [45]. The implemented cTBS protocol has been reported to reduce the excitability of the stimulated brain region for up to 60 minutes, though the stimulation effect decays over time [45]. Performance of the tasks in the post-cTBS session did not take longer than 30 minutes. Accordingly, we could expect the excitability of the stimulated region to be reduced during the full period of task performance in the post-cTBS session, possibly more so at the beginning than the end of the session.

We determined stimulation sites using individual T1-weighted structural scans and Brainsight frameless stereotaxy (Rogue Research). In order to replicate our previous findings on the role of the rTPJ in delaying gratification [11], we used the same coordinates for stimulating the rTPJ (MNI coordinates: x = 60, y = −58, z = 31) as in our previous study. For each participant, we transformed the rTPJ peak coordinates into the native space of the structural scan, using the parameter estimates for spatial normalization of the anatomical scan performed in SPM12. As control site, we used the vertex, which was defined as the meeting point of the pre- and post-central sulcus in the interhemispheric fissure. The cTBS coil was positioned tangentially to the cortical surface over these sites during stimulation, with the handle pointing in a posterior direction.

### fMRI data acquisition and preprocessing

The study was conducted at the Laboratory for Social and Neural Systems Research (SNS lab) at the University Hospital of Zurich. We used a 3-Tesla Philips Achieva whole-body scanner equipped with a 32-channel head coil (Philips Medical Systems, Best, the Netherlands). The functional images were acquired using a BOLD-T2*-weighted single-shot echo-planar imaging (EPI) pulse sequence. The acquisition parameters were as follows: TE = 30 ms; TR = 2,438 ms; flip angle of 75°; FOV = 240 × 216 × 119 mm^2^; acquisition matrix = 80 × 72; voxel size = 3 × 3 × 3 mm^3^; and a slice gap of 1 mm. The anatomical T1-weighted images were obtained after the post-cTBS session using a turbo field echo (TFE) pulse sequence with a flip angle of 8°. The acquisition matrix ranged over 228 × 227 with a field of view (FOV) of 250 × 250 mm^2^ and a voxel size of 1.1 × 1.1 × 0.6 mm^3^.

Preprocessing was performed with SPM 12 (www.fil.ion.ucl.ac.uk/spm). The functional images of each participant were motion corrected, unwarped, slice-timing corrected, and coregistered to the anatomical image. Following segmentation, we spatially normalized the data into standard MNI space. Finally, data were smoothed with an 8-mm FWHM Gaussian kernel and high-pass filtered (filter cutoff = 128 seconds).

### Behavioral data analysis

The statistical analysis of the behavioral data was performed with Matlab R2016b (MathWorks, Natick, MA) and IBM SPSS Statistics 22. We conducted nonparametric Mann-Whitney U tests and MGLMs (using the mixed generalized models module) as implemented in SPSS Statistics 22. As measures of effect size, we computed r(Z/√N) for nonparametric tests. For all analyses, the alpha level was set to 5%. We employed one-tailed, directed tests only for the tests that aimed to replicate our previously reported effect of rTPJ cTBS on temporal discounting [11]; all other tests were two-tailed.

In the intertemporal decision task, we analyzed choice behavior with a model-based approach. We fitted commonly used functions capturing the discounted subjective value of the LL reward as a function of the given delay. Specifically, we modelled choices in the decision task with the hyperbolic discount functions (Eqs 1–3):

$$\begin{array}{ll} SV_{LL}=reward_{LL}\times beta\times delta^{delay} & \left(\text{quasi-hyperbolic}\right) \end{array}$$ (1)

$$\begin{array}{ll} SV_{LL}=\frac{reward_{LL}}{1+k\times delay} & \left(\text{one-parameter}\;\text{hyperbolic}\right) \end{array}$$ (2)

$$\begin{array}{ll} SV_{LL}=\frac{reward_{LL}}{{\left(1+k\times delay\right)}^{s}} & \left(\text{generalized}\;\text{hyperbolic}\right) \end{array}$$ (3)

where SV_LL_ represents the discounted subjective value of the LL reward option and reward_LL_ is the objective amount of the LL reward option. In quasi-hyperbolic discounting [46], beta reflects present bias (intercept of the discount function at delay = 0) and delta the degree of temporal discounting with increasing delay. In one-parameter and generalized hyperbolic discounting [33], k is a parameter that determines the shape of the individual discount function at all delays, whereas generalized hyperbolic discounting adds a scaling factor s that determines the strength of discounting particularly at long delays and that was related to the subjective perception of time [32].

To translate subjective values (as given by Eqs 1–3) into choice, we used a standard softmax function:

$$P(choice\;of\;LL\;option)=\frac{1}{1+e^{-\beta_{temp}\times ({SV}_{LL}-10)}}$$ (4)

This function captures the likelihood of choosing the LL reward option as a function of the difference between the subjective value of the LL reward option (SV_LL_) and the SS reward option (which was fixed to 10 Swiss francs). The free inverse temperature parameter β_temp_ captures how strongly participants relied on this value difference, and temperature can be interpreted as noise. Individual parameters were estimated using a Bayesian approach (2 chains with 4,000 samples; the first 1,000 samples were used as burn-in) based on the algorithms implemented by the hBayesDM package in R [47]. Noninformative flat priors were used for estimation parameters in the pre-cTBS session, whereas in the post-cTBS session we used parameter estimates from the pre-cTBS session as priors. Because the obtained parameter estimates were not normally distributed, we analyzed them with nonparametric Mann-Whitney U tests that assessed cTBS effects (rTPJ versus vertex) on changes in parameter values from pre-cTBS to post-cTBS session (post-cTBS minus pre-cTBS).

In the mental time-travel task, we computed an MGLM that regressed log-transformed RTs on fixed-effect predictors for cTBS (TPJ versus vertex cTBS), session (pre-cTBS versus post-cTBS), run (first versus second run within a session), event type (personal versus non-personal), perspective (now versus future), and event time (relative past versus relative future), as well as all interactions between these factors. The factor run was included to account for the possibility that cTBS effects are transient and might be stronger in the first compared with the second run after stimulation. As random effects, we modelled both participant-specific and event-specific random intercepts as well as slopes for the within-participant predictors session, run, event type, perspective, event time, and all interactions between these factors. Parameter estimates and test statistics were obtained using maximum-likelihood estimation as implemented in SPSS.

### Neuroimaging data analysis

To investigate how rTPJ cTBS modulated neural activation in the intertemporal decision and the mental time-travel task, we used a GLM. This GLM included regressors at option presentation (collapsing over LL and SS reward choices) for the intertemporal decision task. To determine the neural correlates of the subjective value of LL reward, these regressors were modulated by parametric regressors capturing the trial-specific subjective value of the LL reward option as given by the best fitting (generalized) hyperbolic discount function. Moreover, we modeled each motor response with a delta function to account for motor activity. For the mental time-travel task, we included regressors for the 8 distinct trial types in this task (i.e., all combinations of the factors perspective (now versus future), event (personal versus nonpersonal), and relative time (relative past versus relative future). All of these regressors (except the regressor for the motor response) entered the GLM with a duration of 3 seconds. For all models, the regressors were convolved with the canonical hemodynamic response function in SPM. We also added 6 movement (3 translation and 3 rotation) parameters as covariates of no interest.

For statistical analysis of the intertemporal decision task, we first computed for each participant contrasts for subjective LL reward value in the pre-cTBS session (1 for the parametric LL reward regressor in the pre-cTBS session and 0 for all other regressors), in the post-cTBS session (1 for the parametric LL reward regressor in the post-cTBS session and 0 for all other regressors), and for the difference between post-cTBS and the pre-cTBS session (1 for the parametric LL reward regressor in the post-cTBS session, −1 for the parametric LL reward regressor in the pre-cTBS session, and 0 for all other regressors). For the mental time-travel task, please note that the relatively low trial numbers may prevent a reliable analysis of fMRI data. For reasons of completeness, we nevertheless computed individual contrasts reflecting enhanced activation for events in the relative future compared to the relative past in the pre-cTBS session, as well as the difference between post-cTBS and the pre-cTBS session. Imaging results for this task are reported in S3 and S4 Tables, with no effect surviving correction for multiple comparisons. For both tasks, we entered the contrast images from all participants in a between-participant, random effects analysis to obtain statistical parametric maps.

To test the hypothesis that the rTPJ changes the computation of neural value signals in the striatum and VMPFC, we used pre-defined ROIs based on a meta-analysis of temporal discounting [31]. The striatum ROI comprised spheres with diameters of 8 mm (length of spatial smoothing filter) around the peak coordinates in the right (x = 16, y = 2, z = 16) and left striatum (x = −16, y = 14, z = 10). For the VMPFC ROI, we used an 8-mm sphere centered at x = 8, y = 49, z = 10. Based on the same meta-analysis, we also defined a ROI for the DLPFC using an 8-mm sphere around the coordinates x = 42, y = 36, and z = 26. To assess the local specificity of our results, we employed also ROIs for striatum and VMPFC from 2 others studies, one meta-analysis on neural value representations (striatum: x = 12, y = 6, z = −8; VMPFC: x = 0, y = 40, z = −12; [28]) and one canonical study on the neural correlates of discounted delayed reward value (striatum: x = −12, y = 1, z = −8; VMPFC: x = 0, y = 41, z = 8 [14]). We extracted parameter estimates within these ROIs using the Marsbar toolbox for SPM. Because the assumption of normal distribution was violated for several of these extracted parameter estimates (as indicated by significant Kolmogorov-Smirnov tests), we analyzed them with nonparametric tests, but we note that the result pattern is robust to employing parametric tests. In addition, as both the striatum and the VMPFC ROIs test the common hypothesis that rTPJ cTBS affects neural value-coding, we adjusted the alpha level for these comparisons using Bonferroni correction (adjusted alpha: 0.05/2 = 0.025).

In addition to ROI analyses, we conducted exploratory whole-brain second-level analyses for the pre-cTBS session and the difference between post-cTBS and pre-cTBS session (using one-sample *t* tests). For these analyses, we report results outside of our ROIs that survive family-wise error corrections at the peak or cluster level (with a cluster-inducing peak-level threshold of *p* < 0.001, uncorrected) [48]. For the figures only, we set the individual voxel threshold to *p* < 0.005 with a minimal cluster extent of k ≥ 20 voxels. Results are reported using the MNI coordinate system.

### PPI analysis

To examine how rTPJ cTBS modulated the connectivity between the rTPJ and other components of a delay of gratification-enhancing neural network, we conducted a whole-brain PPI analysis with the rTPJ as seed region. We defined the seed region by building a 16-mm sphere (note that the results are robust to using an 8-mm sphere) around the coordinates of the rTPJ cTBS site (MNI coordinates: x = 60, y = −58, z = 31). To create the regressors for the PPI analysis, we first extracted the average time course from the rTPJ seed region for each individual participant (physiological regressor). The subjective value of the LL reward option on each trial (as determined by the generalized hyperbolic discount function) served as (parametric) psychological regressor. We then multiplied the physiological with the psychological regressor and thereby obtained the PPI regressor, which modelled the interaction between the rTPJ time course and the discounted LL reward value.

Next, we computed a GLM (PPI-1) that included the interaction, the physiological, and the psychological regressors. Similar to the activation analysis, we also added onset regressors for the different trial types in the mental time-travel task and onset regressors for choices in the intertemporal decision task, as well as movement parameters as regressors of no interest. We used this model to determine regions in which connectivity with the stimulated rTPJ site was modulated as a function of the subjective LL reward value levels, by calculating the corresponding contrasts (1 for the PPI regressor and 0 for all other regressors) on the single-participant level. These contrasts then entered a second-level analysis to yield statistical parametric maps with a one-sample *t* test.

## Supporting information

S1 Data Excel spreadsheet containing, in separate sheets, the underlying numerical data for Figure panels Figs 2A, 2B, 3A, 3B, 4A, 4B, 5A, 5B, 6A and 6B, S1, S2 and S3 Figs. (XLSX)

S1 Fig Figure showing results of parameter recovery tests for the current data set. (TIFF)

S2 Fig Figure showing results of parameter recovery tests for our previous data set. (TIFF)

S3 Fig Illustration of cTBS effects on value-related activity in rTPJ ROIs. (TIFF)

S1 Text Document containing supporting results. (DOCX)

S1 Table Anatomical locations correlating with discounted delayed reward value in the pre-cTBS session. (DOCX)

S2 Table Anatomical locations showing cTBS effects on functional connectivity with the rTPJ. (DOCX)

S3 Table Anatomical locations correlating with events in the relative future compared to relative past in the mental time-travel task. (DOCX)

S4 Table Anatomical locations for cTBS effects on activation related to relative future compared with relative past events. (DOCX)

## Abbreviations

AUC: area under the curve
cTBS: continuous theta-burst stimulation
DLPFC: dorsolateral prefrontal cortex
EPI: echo-planar imaging
fMRI: functional magnetic resonance imaging
FOV: field of view
GLM: generalized linear model
ITI: inter-trial interval
LL: larger-later
LOOIC: leave-one-out cross-validation information criterion
MGLM: mixed generalized linear model
PPI: psycho-physiological interaction
ROI: region of interest
RT: response time
rTPJ: right temporoparietal junction
SS: smaller-sooner
TFE: turbo field echo
VMPFC: ventromedial prefrontal cortex
