# Supplementary figures and images for: The right temporoparietal junction enables delay of gratification by allowing decision makers to focus on future events
Source: PLoS Biol. 2020 Aug 10;18(8):e3000800. doi: 10.1371/journal.pbio.3000800 (PMC7447039; doi:10.1371/journal.pbio.3000800)

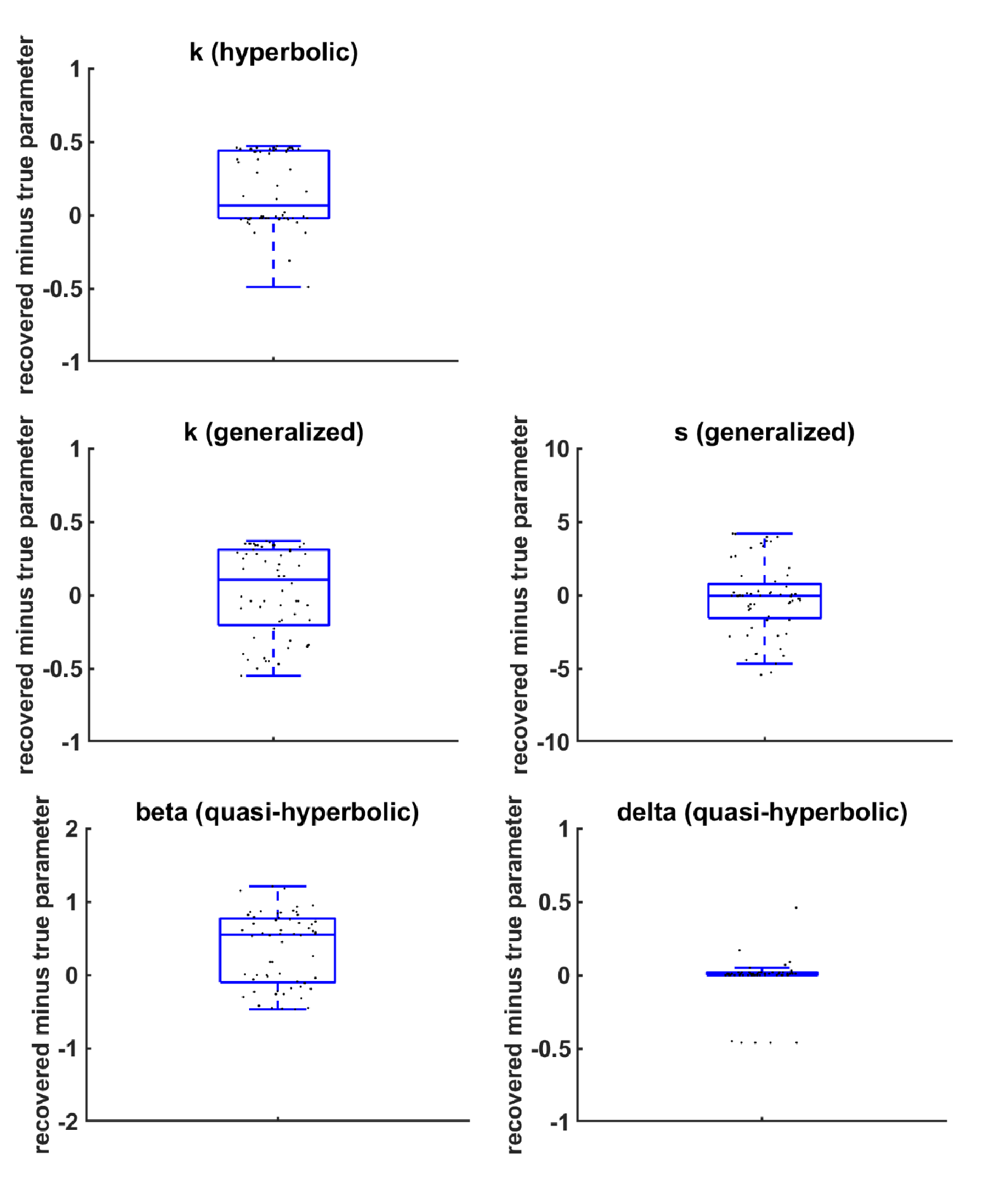

Supplement: S1 Fig — (TIFF) [file pbio.3000800.s002.tiff]

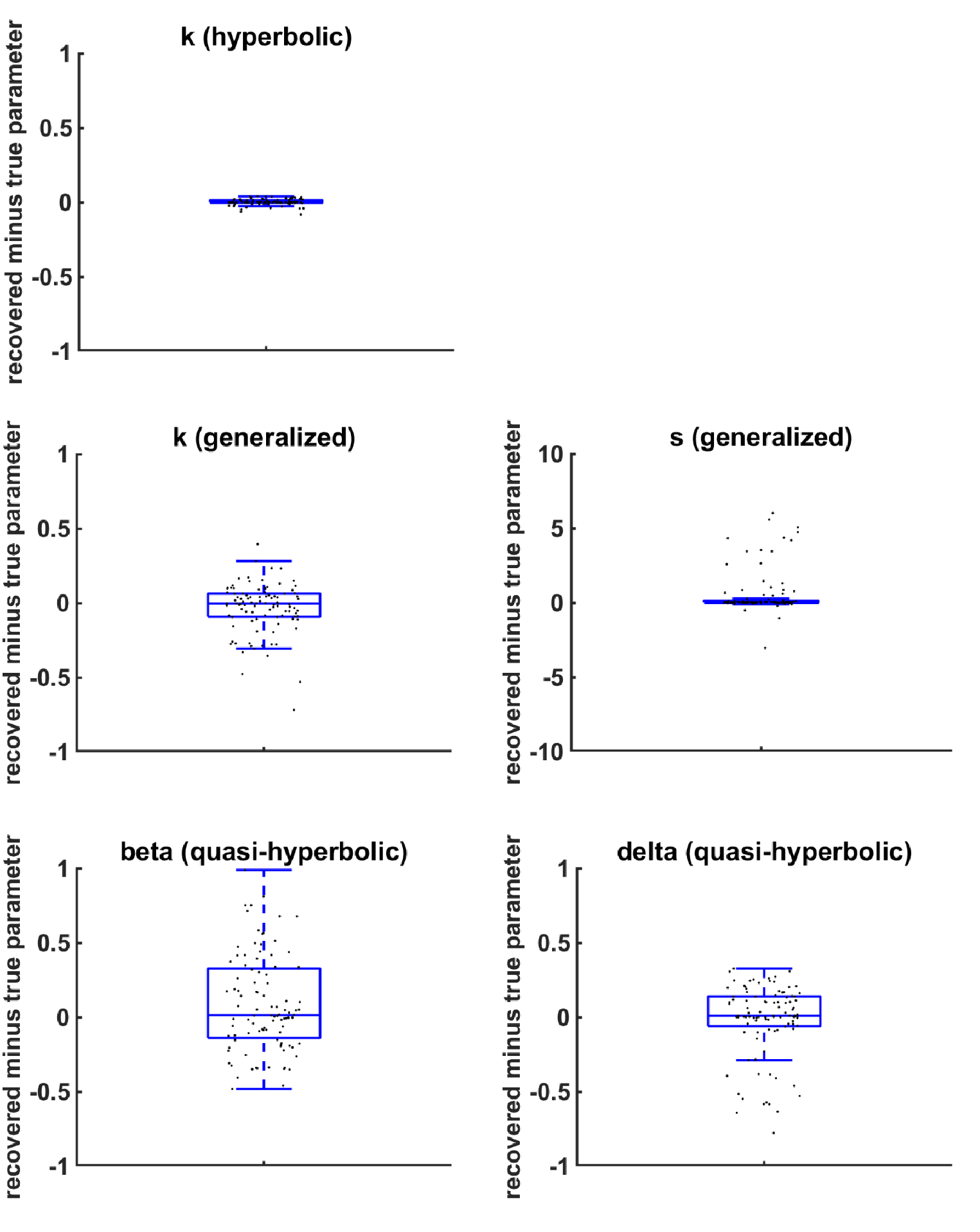

Supplement: S2 Fig — (TIFF) [file pbio.3000800.s003.tiff]

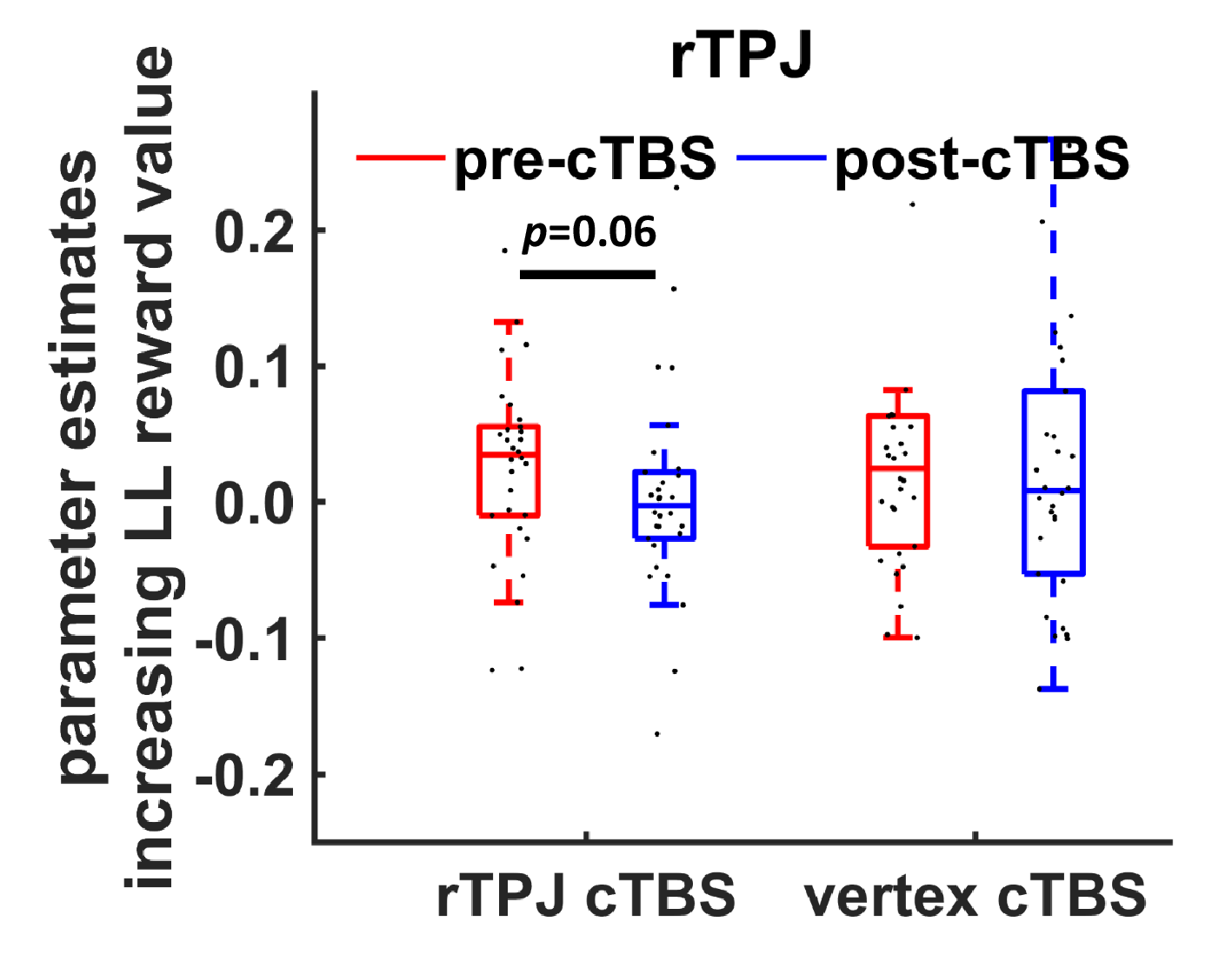

Supplement: S3 Fig — (TIFF) [file pbio.3000800.s004.tiff]
