# Supplementary material for: The right temporoparietal junction enables delay of gratification by allowing decision makers to focus on future events
Source: PLoS Biol. 2020 Aug 10;18(8):e3000800. doi: 10.1371/journal.pbio.3000800 (PMC7447039; doi:10.1371/journal.pbio.3000800)
Supplement: S1 Text — (DOCX) [file pbio.3000800.s005.docx]

**S1_Text**

**The right temporoparietal junction enables delay of gratification by allowing decision makers to focus on future events**

Alexander Soutschek, Marius Moisa, Christian C. Ruff, Philippe N. Tobler

**Supporting results**

*Model and parameter recovery analysis*

We conducted model and parameter recovery analyses for the employed models of delay discounting. The model recovery analysis assessed the specificity with which each model is able to capture data that were generated with this model compared to other models. For this purpose, we first generated artificial data sets (one for each participant, using the same delays and reward magnitudes as in our intertemporal choice task) based on the parameter estimates resulting from fitting each model to the pre-cTBS session. Thus, such simulated data sets were generated separately for the one-parameter, generalized hyperbolic, and quasi-hyperbolic model. We then tested model recovery by fitting all three models to the data sets simulated by the different model types, determining how often (in percentage) the model used to generate the data explained data better (indicated by lower LOOIC) than other models. The data generated by the one-parameter hyperbolic model were better explained by this model compared with the generalized hyperbolic (55%) and the quasi-hyperbolic one (63%). Similarly, the generalized hyperbolic model superseded the one-parameter hyperbolic (80%) and quasi-hyperbolic (82%) models in explaining simulated data created by the generalized hyperbolic model, while the quasi-hyperbolic model outperformed the one-parameter (78%) and generalized hyperbolic (73%) models in explaining the data created by this model. Taken together, the model recovery tests suggest that particularly the generalized hyperbolic and the quasi-hyperbolic models capture distinguishable aspects of delay discounting behavior.

To assess parameter recovery, we computed the difference between the data-generating true and recovered parameters (S1 Fig). Overall, the parameters were recovered fairly accurately with median differences around 0.

*Validation of intertemporal choice results in previous data set*

The cTBS effects on delay discounting conceptually replicate our previous findings [1]. We note though that in our previous study, we had used a different form of a two-parameter hyperbolic discount function with a free intercept parameter in addition to a discount factor (based on a model that originally stemmed from the literature on social discounting and that is not commonly used in delay discounting research). In addition, in our previous study we fitted discount functions to indifference values instead of to binary choices via a softmax link (the latter approach has the advantage of allowing us to consider cTBS effects on decision noise), and in the current study we used a Bayesian approach to parameter estimation. To validate the robustness of our modelling approach, we re-analysed our previous data sets (collapsing across experiment 1 and 2 described in [1]) using the same approach as in our current study (i.e., fitting parabolic, quasi-hyperbolic, and generalized hyperbolic discount functions to binary choice data). This analysis replicates our current findings: in generalized hyperbolic discounting, rTPJ cTBS significantly increased the scaling parameter s, *Z* = 1.80, *p* = 0.04, one-tailed, while leaving k unaffected, *Z* = 0.00, *p* = 1.00. In addition, in one-parameter hyperbolic discounting we observed a significant cTBS effect on k, Mann-Whitney-U test, *Z* = 1.91, *p* = 0.03, one-tailed, and in quasi-hyperbolic discounting there were significant cTBS effects only on delta, *Z* = 2.12, *p* = 0.01, one-tailed, but not beta, *Z* = 0.84 1, *p* = 0.40.This provides further support for the hypothesized causal role of rTPJ for affecting delay discounting particularly at longer delays.

We note that in the old data set, model comparisons favored the one-parameter hyperbolic (mean LOOIC = 84) over the quasi-hyperbolic hyperbolic (mean LOOIC = 106) and the generalized hyperbolic model (mean LOOIC = 106). This discrepancy with the current data set might be explained by differences in the design of the intertemporal choice task. When we again performed model and parameter recovery tests (see above), the one-parameter hyperbolic model clearly outperformed both other models in explaining data generated by this model (in 100% of all cases), and also the quasi-hyperbolic and the generalized hyperbolic model explained data generated by the given model better than other models (in at least 60% of all cases). Parameter recovery tests indicated that the median difference between recovered and true parameters was close to zero for all model parameters (S2 Fig).

*Supplementary ROI analyses*

In addition to the ROI analyses assessing whether cTBS affects value computations in striatum, VMPFC, or DLPFC, we also tested whether value-related activation in the stimulated rTPJ site itself is affected by cTBS. As for all other ROI analyses, we therefore extracted value-related parameter estimates using an 8 mm spherical ROI centered at the coordinates used for rTPJ stimulation (x = 60, y = -58, z = 31). In the pre-cTBS session, rTPJ activation significantly correlated with discounted delayed reward value, *Z* = 2.67, *p* = 0.008. In the rTPJ cTBS group, value-related rTPJ activation tended to be reduced in the post-cTBS relative to the pre-cTBS session, *Z* = 1.59, *p* = 0.06, one-tailed, whereas in the vertex cTBS group rTPJ activation remained unchanged, *Z* < 1, *p* = 0.69. However, there was no evidence that post-cTBS minus pre-cTBS changes were significantly stronger for rTPJ than vertex cTBS, *Z* = 1.02, *p* = 0.32 (S3 Fig). Together with the stimulation effects on connectivity described in the main text, this suggests that rTPJ cTBS effects on functional connectivity between rTPJ and value-coding regions, rather than on rTPJ activation per se, explain the impact of cTBS on delay discounting, consistent with theoretical views on how brain stimulation affects behavior [2].

*S1 Table.* Anatomical locations and MNI coordinates of the peak activations correlating with discounted LL reward value in the pre-cTBS session (GLM-1) at p < 0.001 and a minimum cluster size of k ≥ 10 voxels. Effects surviving whole-brain correction at the cluster level (p < 0.05, family-wise error corrected based on a cluster inducing voxel-level threshold of p<0.001, uncorrected) are in bold.

Hem = Hemisphere (L = left, R = right); BA = Brodmann area

|  |  | |  | | MNI Coordinates | | | | |  | |  |
| --- | --- | --- | --- | --- | --- | --- | --- | --- | --- | --- | --- | --- |
| Region | Hem | | BA | | X | | Y | | Z | k | | t |
| **Fusiform gyrus** | **L** | **37** | | **-60** | | **-49** | | **-1** | | | **204** | **5.23** |
| **Middle frontal gyrus (DLPFC)** | **L** | **8** | | **-30** | | **26** | | **50** | | | **413** | **5.40** |
| **Posterior parietal cortex** | **L** | **39** | | **-48** | | **-64** | | **50** | | | **287** | **4.99** |
|  | **R** | **39** | | **45** | | **-58** | | **47** | | | **172** | **4.85** |
| Medial parietal cortex | L | 7 | | -6 | | -67 | | 62 | | | 11 | 4.57 |
| Temporal cortex | R | 21 | | 66 | | -46 | | 1 | | | 54 | 4.60 |
| Cerebellum | R |  | | 42 | | -73 | | -40 | | | 89 | 4.36 |
| Superior frontal gyrus | R | 8 | | 24 | | 23 | | 59 | | | 73 | 4.26 |
| Cerebellum | R |  | | 15 | | -85 | | -40 | | | 14 | 3.96 |
| Frontopolar cortex | L | 10 | | -39 | | 53 | | -7 | | | 13 | 3.63 |

*S2 Table.* Anatomical locations and MNI coordinates of the peak activations at which rTPJ (relative to vertex) cTBS significantly reduced functional connectivity with the rTPJ as a function of increasing discounted LL reward value (PPI-1) in the post-cTBS compared with the pre-cTBS session (p < 0.001, minimum cluster size k ≥ 10 voxels). No effect survived family-wise error correction at peak or cluster level.

Hem = Hemisphere (L = left, R = right); BA = Brodmann area

|  |  | |  | | MNI Coordinates | | | | |  | |  |
| --- | --- | --- | --- | --- | --- | --- | --- | --- | --- | --- | --- | --- |
| Region | Hem | | BA | | X | | Y | | Z | k | | t |
| Occipital cortex | R | 18 | | 6 | | -70 | | -10 | | | 20 | 3.71 |

*S3 Table.* Anatomical locations and MNI coordinates of the peak activations correlating with events in the relative future compared to relative past in the pre-cTBS session of the mental time travel task (GLM-1) at p < 0.001 and a minimum cluster size of k ≥ 10 voxels. No effect survived whole-brain correction at the cluster level (p < 0.05, family-wise error corrected based on a cluster inducing voxel-level threshold of p<0.001, uncorrected).

Hem = Hemisphere (L = left, R = right); BA = Brodmann area

|  |  | |  | | MNI Coordinates | | | | |  | |  |
| --- | --- | --- | --- | --- | --- | --- | --- | --- | --- | --- | --- | --- |
| Region | Hem | | BA | | X | | Y | | Z | k | | t |
| Cerebellum  Parietal cortex | R  L  R | 39 | | 9  -9  45 | | -55  -55  -55 | | -43  -43  56 | | | 48  21  27 | 4.01  3.96  3.38 |
| Dorsolateral prefrontal cortex | R | 8 | | 42 | | 11 | | 44 | | | 15 | 3.27 |

*S4 Table.* Anatomical locations and MNI coordinates of the peak activations where post-cTBS minus pre-cTBS changes in activation related to relative future compared with relative past events are stronger in the rTPJ cTBS group than in the vertex cTBS group in the mental time travel task (p < 0.001 and a minimum cluster size of k ≥ 10 voxels). No effect survived whole-brain correction at the cluster level (p < 0.05, family-wise error corrected).

Hem = Hemisphere (L = left, R = right); BA = Brodmann area

|  |  | |  | | MNI Coordinates | | | | |  | |  |
| --- | --- | --- | --- | --- | --- | --- | --- | --- | --- | --- | --- | --- |
| Region | Hem | | BA | | X | | Y | | Z | k | | t |
| Inferior frontal gyrus | L | 45 | | -42 | | 23 | | 8 | | | 20 | 3.55 |

*S1 Fig.* Results of parameter recovery tests for the current data set. The median differences between true and recovered parameters are close to zero for all parameter estimates. The underlying data for this Figure can be found in S1_Data.

*S2 Fig.* Results of parameter recovery tests for our previous data set. The median differences between true and recovered parameters are close to zero for all parameter estimates. The underlying data for this Figure can be found in S1_Data.

*S3 Fig.* Illustration of cTBS effects on value-related activity in rTPJ ROIs. While value-related parameter estimates tended to be reduced in the post-cTBS relative to the pre-cTBS session in the rTPJ cTBS group, this effect was not significantly stronger compared with the vertex cTBS group. Boxes indicate median (in red) and interquartile range. Black dots show individual parameter estimates. The underlying data for this Figure can be found in S1_Data.

**References**

1. Soutschek A, Ruff CC, Strombach T, Kalenscher T, Tobler PN. Brain stimulation reveals crucial role of overcoming self-centeredness in self-control. Science advances. 2016;2(10):e1600992. doi: 10.1126/sciadv.1600992. PubMed PMID: 27774513.

2. Ruff CC, Driver J, Bestmann S. Combining TMS and fMRI: from ‘virtual lesions’ to functional-network accounts of cognition. Cortex. 2009;45(9):1043-9.
