# Supplementary material for: The right temporoparietal junction enables delay of gratification by allowing decision makers to focus on future events
Source: PLoS Biol. 2020 Aug 10;18(8):e3000800. doi: 10.1371/journal.pbio.3000800 (PMC7447039; doi:10.1371/journal.pbio.3000800)
Supplement: S1 Table — (DOCX) [file pbio.3000800.s006.docx]

|  |  | |  | | MNI Coordinates | | | | |  | |  |
| --- | --- | --- | --- | --- | --- | --- | --- | --- | --- | --- | --- | --- |
| Region | Hem | | BA | | X | | Y | | Z | k | | t |
| **Fusiform gyrus** | **L** | **37** | | **-60** | | **-49** | | **-1** | | | **204** | **5.23** |
| **Middle frontal gyrus (DLPFC)** | **L** | **8** | | **-30** | | **26** | | **50** | | | **413** | **5.40** |
| **Posterior parietal cortex** | **L** | **39** | | **-48** | | **-64** | | **50** | | | **287** | **4.99** |
|  | **R** | **39** | | **45** | | **-58** | | **47** | | | **172** | **4.85** |
| Medial parietal cortex | L | 7 | | -6 | | -67 | | 62 | | | 11 | 4.57 |
| Temporal cortex | R | 21 | | 66 | | -46 | | 1 | | | 54 | 4.60 |
| Cerebellum | R |  | | 42 | | -73 | | -40 | | | 89 | 4.36 |
| Superior frontal gyrus | R | 8 | | 24 | | 23 | | 59 | | | 73 | 4.26 |
| Cerebellum | R |  | | 15 | | -85 | | -40 | | | 14 | 3.96 |
| Frontopolar cortex | L | 10 | | -39 | | 53 | | -7 | | | 13 | 3.63 |
