# Supplementary material for: The right temporoparietal junction enables delay of gratification by allowing decision makers to focus on future events
Source: PLoS Biol. 2020 Aug 10;18(8):e3000800. doi: 10.1371/journal.pbio.3000800 (PMC7447039; doi:10.1371/journal.pbio.3000800)
Supplement: S2 Table — (DOCX) [file pbio.3000800.s007.docx]

|  |  | |  | | MNI Coordinates | | | | |  | |  |
| --- | --- | --- | --- | --- | --- | --- | --- | --- | --- | --- | --- | --- |
| Region | Hem | | BA | | X | | Y | | Z | k | | t |
| Occipital cortex | R | 18 | | 6 | | -70 | | -10 | | | 20 | 3.71 |
