# Supplementary material for: The right temporoparietal junction enables delay of gratification by allowing decision makers to focus on future events
Source: PLoS Biol. 2020 Aug 10;18(8):e3000800. doi: 10.1371/journal.pbio.3000800 (PMC7447039; doi:10.1371/journal.pbio.3000800)
Supplement: S3 Table — (DOCX) [file pbio.3000800.s008.docx]

|  |  | |  | | MNI Coordinates | | | | |  | |  |
| --- | --- | --- | --- | --- | --- | --- | --- | --- | --- | --- | --- | --- |
| Region | Hem | | BA | | X | | Y | | Z | k | | t |
| Cerebellum  Parietal cortex | R  L  R | 39 | | 9  -9  45 | | -55  -55  -55 | | -43  -43  56 | | | 48  21  27 | 4.01  3.96  3.38 |
| Dorsolateral prefrontal cortex | R | 8 | | 42 | | 11 | | 44 | | | 15 | 3.27 |
