# Supplementary material for: The right temporoparietal junction enables delay of gratification by allowing decision makers to focus on future events
Source: PLoS Biol. 2020 Aug 10;18(8):e3000800. doi: 10.1371/journal.pbio.3000800 (PMC7447039; doi:10.1371/journal.pbio.3000800)
Supplement: S4 Table — (DOCX) [file pbio.3000800.s009.docx]

|  |  | |  | | MNI Coordinates | | | | |  | |  |
| --- | --- | --- | --- | --- | --- | --- | --- | --- | --- | --- | --- | --- |
| Region | Hem | | BA | | X | | Y | | Z | k | | t |
| Inferior frontal gyrus | L | 45 | | -42 | | 23 | | 8 | | | 20 | 3.55 |
